# Evaluation of clinical and paraclinical effects of intraosseous vs intravenous administration of propofol on general anesthesia in rabbits 

**Published:** 2012

**Authors:** Ramin Mazaheri-Khameneh, Farshid Sarrafzadeh-Rezaei, Siamak Asri-Rezaei, Bahram Dalir-Naghadeh

**Affiliations:** *Department of Clinical Sciences, Faculty of Veterinary Medicine, Urmia University, Urmia, Iran.*

**Keywords:** Propofol, Blood profile, Intraosseous injection, Anesthesia, Rabbit

## Abstract

This prospective study aimed to compare the intraosseous (IO) and intravenous (IV) effects of propofol on selected blood parameters and physiological variables during general anesthesia in rabbits. Thirty New Zealand White rabbits were studied. Six rabbits received IV propofol (group 1) and another 6 rabbits, were injected propofol intraosseously (Group 2) for 30 minutes (experimental groups). Rabbits of the third and fourth groups received IV and IO normal saline at the same volume given to the experimental groups, respectively. In the fifth group IO cannulation was performed but neither propofol nor normal saline were administered. Blood profiles were assayed before induction and after recovery of anesthesia. Heart and respiratory rates, rectal temperature, saturation of peripheral oxygen and mean arterial blood pressure were recorded. Heart rate increased significantly 1 to 5 minutes after induction of anesthesia in experimental groups (*P* < 0.05). Although mean arterial blood pressure decreased significantly from baseline, values remained above 60 mm Hg (*P* < 0.05). Respiratory rate decreased significantly in experimental groups, but remained higher in group 2 (*P* < 0.05). The lymphocyte count decreased significantly in group 1 (*P* < 0.05). The concentration of alkaline phosphatase in all rabbits, aspartate aminotransferase and gamma-glutamyl transferase in the first group and gamma-glutamyl transferase in the third group increased significantly (*P* < 0.05). Total bilirubin decreased significantly in group 2 (*P* < 0.05). All measured values remained within normal limits. Based on the least significant physiological, hematological and biochemical effects, the IO injection of propofol appears to be safe and suitable method of anesthesia in rabbits with limited vascular access.

## Introduction

Rabbits are the third most commonly anesthetized species, but have at least seven times more risks of anesthetic-related death compared to dogs and cats.^1^ Currently, advanced diagnostic and surgical procedures requiring safe and adequate anesthesia are routinely performed on rabbits.^2^ Tracheal intubation of rabbits and use of inhalation anesthetic can be quite complicated and time consuming.^3^ Intubation can cause trauma to the larynx, laryngospasm and tracheal lesions,^4^ so requires experience and technical dexterity. In small and laboratory animals, most anesthetics are administered intramuscularly because of the difficulty in obtaining intravenous (IV) access. The introduction of short acting hypnotic drugs like propofol prompted the development of alternative methods to inhalation anesthesia, i.e. total intravenous anesthesia (TIVA).^5^

Propofol, a non-barbiturate substituted isopropyl phenol is an IV anesthetic currently popular in human beings and animals as both a sole agent and as an adjunct in balanced anesthetic techniques.^6^ It has been used successfully to induce anesthesia in rabbits at doses of 5-15 mg kg^-1^ of body weight.^7^^-^^9^ The major disadvantage of propofol is the need for IV administration. Other routes of administration such as the intraperitoneal or intramuscular route have no anesthetic effects at the doses investigated.^8^

The intraosseous (IO) route is effective for the administration of many drugs that induce chemical restraint.^1^^-^^3^^,^^10^ and the technique has almost completely replaced venous cut-down procedures in children, adults and animals.^11^^-^^13^ When rapid venous catheterization is impossible the IO route has potential advantages in the induction and maintenance of general anesthesia with proper anesthetics. While considerable information on the pharmacokinetic, pharmacodynamic and hematological effects of propofol are available,^8^^,^^14^ its IO effects on hematological variables have not yet been studied.

The aim of this study was to compare the IO and IV effects of propofol on selected physiological and hematological variables during the induction and maintenance of anesthesia with propofol in rabbits.

## Materials and methods

This study was approved by the ethics committee for Studies on Laboratory Animals of the Department of Clinical Sciences Review Board at the Faculty of Veterinary Medicine of Urmia University, Urmia, Iran. Thirty male New Zealand White rabbits, weighing 2.4 ± 0.5 kg, were examined before study and found to be in good health. They were kept in a controlled environment with a temperature of 20 ± 2 °C and had free access to commercial pellet diet (Niro-Sahand Co., Tabriz, Iran) and water until 1 hour before induction of anesthesia. Rabbits were divided into two experimental and three control groups, each containing six. In all of the rabbits the skin of the dorsal base of one ear was infiltrated with 0.5-1 mL of lidocaine hydrochloride 1% solution (Kela Laboratoria, Hoogstraten, Belgium) to induce vasodilatation of the ear vessels to facilitate arterial and venous cannulation. Ten minutes later the central auricular artery and a marginal ear vein were cannulated with a 22 gauge over-the-needle catheter (Mediplus India Ltd., Haryana, India) for monitoring blood pressure, blood sampling and IV drug administration. The rabbits were restrained manually during IV and IO cannulation. The latter was accomplished by aseptically preparing the cranio-medial aspect of the tibia plateau before desensitizing the skin and periosteum with 0.5 mL of lidocaine hydrochloride 1% solution. A 22 gauge, 3 cm long bone marrow aspiration needle was inserted into the medullary cavity and the stylet removed before the needle was flushed with heparinized saline (10 IU mL^-1^) solution and the needle secured in place. Rabbits were allowed to rest for 15 minutes after venous and arterial catheterization or osseous cannulation before pre-anesthetic data were collected. 

The respiratory rate (RR) was determined by direct observation and heart rate (HR), saturation of peripheral oxygen (SpO_2_) and rectal temperature were measured before induction of anesthesia, during the first 5 minutes and then at five-minute intervals throughout the anesthetic period directly by patient Monitor (General Meditech Inc, Shenzhen, China). The catheter of central auricular artery was connected to physiological pressure transducer (AD Instruments, Chalgrove, UK), data acquisition system (AD Instrument, Sydney, Australia) to enable monitoring of the direct mean arterial pressure (MAP) which was calculated as MAP = diastolic BP+0.3 (systolic BP–diastolic BP).^8^ All rabbits breathed room air spontaneously during the study.

In group 1, propofol 1% (12.5 mg kg^-1^, B. Braun, Melsungen AG, Germany) was administered IV over 10 seconds for induction of anesthesia. Anesthesia was then maintained for 30 minutes by a constant rate IV propofol infusion (1 mg kg^-1^ min^-1^)^9^ using an automated delivery system (B. Braun Schiwa GmbH & Co. KG, Glandorf, Germany). In group 2, the dose and procedure for propofol administration was as for group 1 except that the IO route was used. The third group of animals was prepared as for group 1 but normal saline, was given. In group 4 normal saline was administered IO in the same way as propofol was administered in group 2. In group 5 only IO tibial cannulation was performed without administration of neither propofol nor normal saline.

Induction time was defined as the interval from administration of propofol to loss of righting reflex. Propofol anesthesia was discontinued after 30-minutes and recovery time was recorded. Anesthesia time was defined as the interval from loss of the righting reflex to its return. Quality of anesthesia was evaluated according to the following anesthesia scale: 0= Severe reaction with violent movement; 1= Moderate reaction, able to stay in sternal position and mildly responsive to external stimuli (such as sound and hand waving); 2= Unable to stay in sternal recumbency and tending to stay in lateral recumbency. Hardly response to pinna and pedal withdrawal reflexes; 3= Deep general anesthesia, no reaction. All of reflexes were absent except corneal reflexes with mild reaction to stimuli. Recovery time was defined as a period between the end of propofol administration and returning of the righting reflex. Quality of recovery from induction to return of the righting reflex was evaluated by use of the following scale: 0 = Quite and smooth; 1 = Occasional thrashing and violent movement; 2 = Constant thrashing and violent movement; 3 = No effect.

Blood hematology and plasma chemistry profiles were compared in rabbits before propofol administration and after recovery from anesthesia and among rabbits of different groups on each occasion. An arterial blood sample of 2 mL was drawn into glass vials containing 1.0 mg mL^-1^ ethyldiamine tetracetic acid (EDTA) to determine the hematological component according to the method of Mitruka and Rawnsley.^15^ Packed cell volume (PCV), was measured by microhematocrit tubes. Hemoglobin (Hb) was determined by the cyanmethemoglobin method. Total red blood cell (RBC) and white blood cell (WBC) counts were determined using an improved Neubauer hemocyto-meter method.^16^ Blood smears were air-dried and stained using Giemsa-Romanowski stain. Two hundred leukocytes were counted for each smear and classified as neutrophils (heterophils), eosinophils, basophils, lymphocytes and monocytes. Another 2 mL blood sample was allowed to clot in glass vials without coagulant and centrifuged at 3000 *g* for 20 minutes at 4°C. The serum was removed, frozen (-20 °C) and analyzed to determine the biochemical components. The serum was analyzed using an automated analyzer (Technicon, RA1000, USA) for total protein (TP), glucose (Glu), urea, alkaline phosphatase (ALP), alanine aminotransferase (ALT), aspartate aminotransferase (AST), gamma-glutamyl transferase (GGT), creatinine (Cre), total bilirubin (TB), cholesterol (Chol), triglycerides (TG), phosphorus (P) and calcium (Ca). 

Statistical analyses were performed using SAS version 9.1 (SAS Institute, Inc., Cary, USA). Data were analyzed as repeated measures using mixed models. The included fixed effects were treatment (route of administration), repeated effect of time, and their interaction term. The random effect was animals nested within treatments. For each parameter five covariance structures CS, UN, SP (POW), SP (GAU), and SP (SPH) were evaluated. The appropriate correlation structure was determined by identifying the smallest Akaike’s information criterion. SP (POW) and SP (GAU) structures provided the best model fit for the parameters. LSMEANS statement was used to calculate the least-squares means and standard errors. When the effects were significant, pair-wise differences between the least-squares means were compared using the PDIFF-option and Tukey's adjustment *t*-test in the LSMEANS statement. For each model, the Shapiro-Wilks test and examination of histograms and residual plots were used to explore the assumption of normality and homogeneity of variation. When the assumption of normality and/or homogeneity of variance were rejected, the data were transformed using the natural logarithm or square roost to obtain normality or homogeneity of variation of the residuals. The level of significance was set at *P* < 0.05. Data are presented as least-square means ± standard error of means (SEM). 

## Results

Induction of and recovery from anesthesia were uneventful in all experimental groups. Animals in groups 4 and 5 tolerated bone marrow cannulation under local anesthesia. The data for induction, anesthetic, and recovery time are presented in table 1. Statistical analysis showed no significant difference between anesthetic times in these groups (*P* > 0.05). All rabbits had a smooth, excitement-free and quiet recovery (Table 1). 

**Fig. 1:**
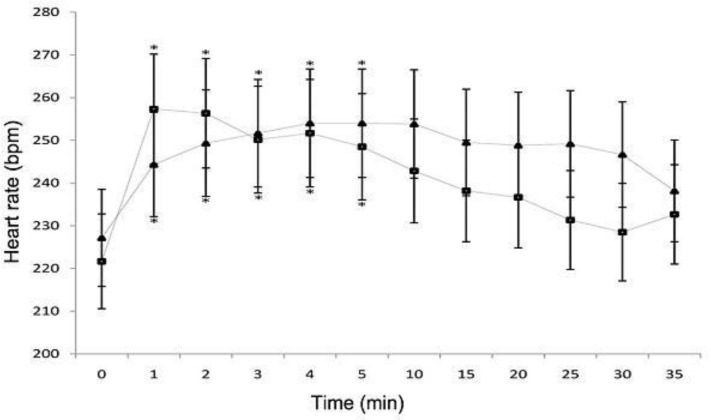
Mean (±SEM) heart rate (bpm: beats per minute) of rabbits given propofol IV (Group 1 [triangles]) and IO (Group 2 [squares]) for induction and maintenance of anesthesia in experiment groups. * Significantly (*P* < 0.05) different from value at time 0.

**Fig. 2:**
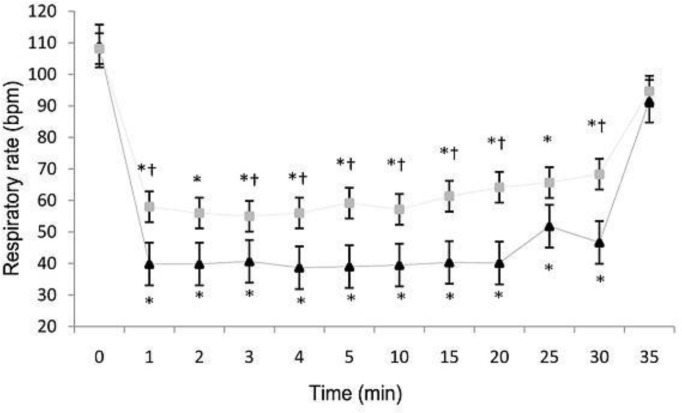
Mean (±SEM) respiratory rate (bpm: breaths per minute) of rabbits given propofol IV (Group 1 [triangles]) and IO (Group 2 [squares]) for induction and maintenance of anesthesia in experiment groups. * Significantly (*P* < 0.05) different from value at time 0. ^†^ Significantly (*P* < 0.05) different between group 1 and group 2 groups

There were no significant differences (*P* > 0.05) between groups 1 and 2 in mean HR, MABP, SpO_2_ and body temperature at any given time. Mean baseline HR were 227.00 ± 4.27 bpm (beats per minute) for group 1 animals and 221.00 ± 4.27 bpm for those in group 2. Elevated HR was significant only 1 to 5 minutes after induction of anesthesia in both groups (Fig. 1). A significant decrease in mean RR (*P* < 0.05) was noted from 1 to 30 minutes during propofol anesthesia in each group, but RR were significantly high in group 2 at 1, 3, 4, 5, 10, 15, 20, and 30-minutes after induction compared to that of rabbits anesthetized by the IV route (*P* < 0.05) (Fig. 2). 

**Fig. 3:**
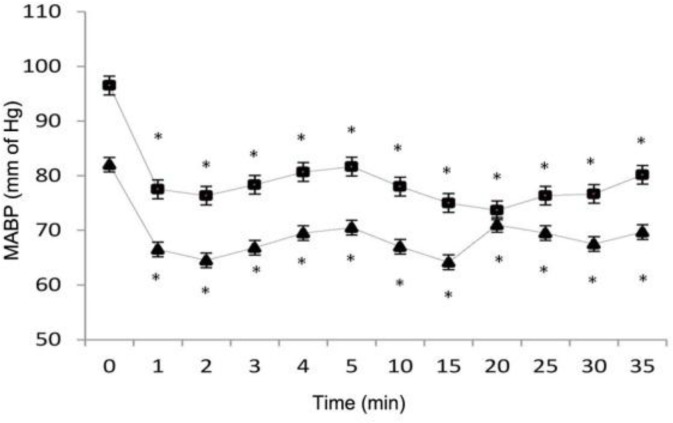
Mean (±SEM) arterial blood pressure values of both experiment groups given propofol IV (Group 1 [triangles]) and IO (Group 2 [squares]) for induction (12.5 mg kg^-1^) and maintenance (1 mg kg^-1^ min^-1^) of anesthesia. * Significantly (*P* < 0.05) different from value at time 0

The MABP and mean SpO_2_ values decreased significantly compared to baseline value (*P* < 0.05). The MABP remained above 60 mm of Hg and pale mucous membranes were not observed in rabbits anesthetized by IV or IO propofol. These changes were significant for 30 minutes after induction in both groups (Fig. 3).

**Table 1:** The quality of anesthesia and recovery, time of induction, duration of anesthesia and time of recovery in rabbits receiving propofol IV and IO. Data are presented as mean ± SEM

Parameters	Group 1 (IV)	Group 2 (IO)
Time to induction (sec)	9.00 ± 0.81	8.00 ± 0.45
Time of anesthesia (min)	37.00 ± 0.96	38.00 ± 0.73
Time to recovery (min)	7.75 ± 0.98	9.00 ± 0.85
Quality of anesthesia	2	2
Quality of recovery	0	0

No significant differences were detected between anesthetic times between groups (*P* > 0.05).

The hematological and biochemical values obtained for rabbits before and after anesthesia in the experimental groups or any other interventions in the control groups are presented in Tables 2 and 3. Significant differences (*P* < 0.05) were found only in the concentration of lymphocytes in group 1 (Table 2). Packed cell volume values decreased in all groups, but this was not significant compared to the baseline values. There were also no significant decreases in Hb concentration, PCV and RBC. All hematological parameters were within the normal range after treatments in rabbits of the experimental and control groups. The WBC count increased after anesthesia although there were no significant differences between pre-and post-anesthetic values. The blood plasma was characterized by a significant increase in ALP among all rabbits (*P* < 0.05). AST and GGT values were significantly increased in group 1 (*P* < 0.05). Increased GGT activity was noted in group 3 (*P* < 0.05). The total bilirubin concentration decreased significantly in group 2 only (*P* < 0.05). All biochemical parameters remained within normal limits (Table 3).

**Table 2:** Selected blood hematological parameters (SI Unit) in rabbits before and after drug administration. Values are presented as mean ± SEM.

**Groups**	**Time**	**Hb ** **(mmol L** ^-1^ **)**	**PCV ** **(L L** ^-1^ **)**	**RBC ** **(×10** ^12^ ** L** ^-1^ **)**	**WBC ** **(×10** ^9^ ** L** ^-1^ **)**	**Neutrophil ** **(×10** ^9^ ** L** ^-1^ **)**	**Eosinophil ** **(×10** ^9^ ** L** ^-1^ **)**	**Basophil ** **(×10** ^9^ ** L** ^-1^ **)**	**Monocyte ** **(×10** ^9^ ** L** ^-1^ **)**	**Lymphocyte ** **(×10** ^9^ ** L** ^-1^ **)**
1	Before	8.26	0.39	5.90	8.70	3.56	0.05	0.17	0.20	4.67
	After	7.54	0.36	5.10	11.40	6.49	0.13	0.24	0.19	3.81*
2	Before	8.64	0.41	5.50	8.20	3.51	0.09	0.19	0.16	4.22
	After	8.34	0.40	4.80	10.50	5.90	0.17	0.21	0.21	3.97
3	Before	7.78	0.37	5.80	8.60	2.56	0.07	0.01	0.14	5.80
	After	7.26	0.35	5.60	8.80	2.92	0.08	0.00	0.26	5.50
4	Before	7.15	0.35	5.50	9.60	3.54	0.16	0.00	0.24	5.54
	After	6.82	0.34	5.40	9.90	4.20	0.24	0.00	0.28	5.21
5	Before	7.59	0.36	5.60	8.20	3.14	0.08	0.00	0.16	4.78
	After	7.40	0.35	5.60	7.50	2.84	0.10	0.00	0.17	4.37
	SEM	0.23	0.01	0.28	0.63	0.02	0.00	0.00	0.00	0.01

* Significantly different from value at the time before drug administration (*P* < 0.05).

**Table 3:** Selected serum biochemical parameters (SI Unit) in rabbits before and after drug administration. Values are presented as mean ± SEM

**Group**	**Time**	**TP ** **(g L** ^-1^ **)**	**Glu ** **(mmol L** ^- 1^ **)**	**Urea** **(mmol L** ^-1^ **)**	**ALP ** **(U L** ^-1^ **)**	**ALT ** **(U L** ^-1^ **)**	**AST ** **(U L** ^-1^ **)**	**GGT ** **(U L** ^-1^ **)**	**Cre ** **(µmol L** ^-1^ **)**	**TB ** **(µmol L** ^-1^ **)**	**Chol ** **(mmol L** ^-1^ **)**	**TG ** **(mmol L** ^-1^ **)**	**Ca ** **(mmol L** ^-1^ **)**	**P ** **(mmol L** ^-1^ **) **
1	Before	63.40	6.20	12.39	207.10	28.80	32.70	9.51	121.99	2.12	1.37	1.79	2.22	1.30
	After	59.00	6.10	12.01	222.17*	32.42	35.38*	11.81	108.73	1.96	1.45	1.74	2.46	1.25
2	Before	64.50	6.16	14.55	220.00	33.34	37.50	12.26	113.15	3.16	1.41	1.71	2.51	1.28
	After	61.50	6.21	16.40	239.44*	36.44	40.25	12.63	126.41	1.84*	1.38	1.86	2.71	1.50
3	Before	68.00	6.10	10.53	191.55	30.61	33.14	9.92	114.92	3.04	1.45	1.53	2.31	1.26
	After	69.50	6.63	11.73	201.80*	30.71	33.72	12.24	118.45	3.58	1.42	1.82	2.38	1.35
4	Before	67.00	6.41	10.79	211.20	32.55	34.90	11.00	106.96	2.61	1.50	1.60	2.48	1.39
	After	66.20	6.50	10.95	228.24*	32.73	36.80	12.55	102.54	2.90	1.45	1.64	2.49	1.45
5	Before	68.20	5.83	13.11	205.06	29.90	34.77	9.97	108.73	2.06	1.33	1.63	2.38	1.36
	After	65.10	6.42	13.29	216.22*	33.26	36.61	11.15	108.73	1.71	1.40	1.53	2.63	1.37
	SEM	2.20	0.18	0.75	7.74	1.36	1.33	0.53	5.21	0.22	0.08	0.08	0.08	0.07

* Significantly different from value at the time before drug administration (*P* < 0.05).

## Discussion

Veterinary anesthesia demands increasingly safer techniques which could enable rapid recoveries and low incidence of adverse effects.^17^^,^^18^ In the present study the anesthetic and the pharmacodynamics effects of IO propofol was compared with that of its IV administration. This study demonstrated that the IO route was as effective as the IV route for propofol administration at doses inducing general anesthesia. The rate of absorption by the IO route was rapid and not different from IV injection, as revealed by the rapid induction. Similar findings have been described by other authors.^2^^,^^3^^,^^10^^-^^12^^,^^19^ Cardiovascular and respiratory effects of propofol administration in rabbits, dogs, rats, sheep and horse include heart rate elevation, post-induction apnea, hypoventilation, hypotension and decreased oxygen saturation.^6^^,^^20^^-^^25^ A significant tachycardia persisted for 5 minutes in the experimental groups (Fig. 1). The cause of increased HR during propofol anesthesia is not clear.^20^ Since MABP decreased significantly in group 1 and 2 rabbits, the tachycardia was probably a reflex response to hypotension.^26^ Generally, arterial vasodilatation and venodilatation, caused by reduction in sympathetic nervous outflow and a direct relaxant effect on vascular smooth muscle explains the lower blood pressures recorded during anesthesia.^21^^,^^22^^,^^27^^-^^30^

Although significant decreases in SpO_2_ values were recorded, cyanotic mucous membranes or ears were not observed; this may have been related to MABP values that persisted > 60 mmHg throughout anesthesia.^8^ In groups 1 and 2, the decrease in RR was significant from 1 to 30 minutes during anesthesia in both groups. However, the RR remained significantly higher in group 2 animals compared to those in group 1 (Fig. 2), a result which was also seen when thiopental, rather than propofol was used.^3^

The IV and IO administration of fluids also affect hematological profile.^31^ The foremost purpose of this study was to examine the effects of an IO administration of propofol on hematologic and biochemistry indexes in arterial blood samples. The 30-minute anesthesia of twelve rabbits using propofol IV or IO was safe. All hematological and biochemical parameters remained within the physiological range of healthy individuals.^32^^-^^35^

In sheep, all hematological parameters have been reported to be within the physiological limits during propofol anesthesia.^36^ A decrease in PCV, Hb and RBC count values and an increase in WBC count values after surgery performed under acetylpromazine–propofol anesthesia in dogs have been reported.^37^ It is known that propofol induces moderate systemic hypotension, arterial vasodilatation and venodilatation.^40^ In the present study there were no significant differences in PCV, Hb, RBC and WBC values before and after administration of propofol in experimental groups. These values also remained unchanged in the control groups. The slight rise in the WBC concentration was evident in animals of the all groups of the current study. Prolonged periods of stress cause neutrophilia and lymphopaenia.^41^ In a study, marked changes in white blood cell distribution with a relative neutrophilia and lymphopenia were found in rabbits during air or lorry transport which was in direct relationship with cortisol levels of corresponding stress.^41^ The mild neutrophilia and lymphopenia in all groups of the current study with a marked lymphopenia observed in group 1 rabbits could be explained as a reaction to the stress arising from manual handling and restraint and the cannulation of the auricular veins and arteries. The marked lymphopenia was only seen in group 1 rabbits, whereas this change was not significant in group 2. Eosinophil, basophil and monocyte counts were unaltered during the peri-anesthesia period, even in control groups. 

Total protein, blood urea and serum creatinine values did not show any significant differences between treatment groups. As in other species, an elevated blood urea value in rabbits is associated with renal insufficiency. Low blood urea levels can also reflect hepatic dysfunction. Any changes in blood creatinine concentrations are due to changes in excretion and reflect renal function.^42^

Hypercalcemia is seen in rabbits with chronic renal failure and impaired calcium excretion. Hyperphosphatemia can also result from impaired renal phosphorus excretion due to kidney disease. In the present study levels of total blood calcium or phosphorus after recovery of anesthesia in experimental or after described interventions in control groups remained unchanged and were within the normal range. These results may reflect the safety of IO or IV administration of propofol in relation to liver and kidney functions. In rabbits, hepatic ALT activity is lower than in other species and there is less organ specificity.^43^ The ALT remained within the clinical normal range after recovery and after drug infusion in all groups. In rabbits, AST is found in the liver, heart, skeletal muscle, kidney and pancreas with the highest activity in the liver and skeletal muscle.^44^ Physical exertion or tissue damage during blood collection can elevate results. Raised AST levels can be found in association with liver disease. Mild raised AST levels were seen after anesthesia in the experimental groups; however, it was significant only in group 1. A slight increase in AST values is reported in dogs when anesthetized with methotrimeprazine-propofol.^45^

The liver is the predominant site for propofol glucuronidation in most species, although extra-hepatic glucuronidation in kidney and gastrointestinal tissues has been documented in several species.^46^ Hepatic metabolism results in inactive, water-soluble sulfate and glucuronic acid metabolites that are excreted by the kidneys.^14^^,^^47^

In rabbit GGT is located predominantly in the renal epithelium with low activity in the liver. Liver GGT is present primarily in bile duct epithelial cells and is therefore an indicator of hepatobiliary disease rather than hepatocellular damage.^48^ Atropine-medetomidine-propofol raises GGT activity levels in dogs.^49^ In rabbits, ALP is present in nearly all tissues, in association with cell membranes, and especially in intestinal epithelium, renal tubules, osteoblasts, liver and placenta. In the present study a marked rise in ALP values was seen in animals of all groups (*P* < 0.05); the concentration of GGT was significantly higher in groups 1 and 3. Despite these, the values remained within the normal range. The unexpected ALP and GGT elevations could be explained in terms of the hepatic metabolism of propofol and the renal excretion of its metabolites by the kidneys. It is possible that the increased activity of ALP and AST resulted from restraint or various attempts at venous and arterial puncture. Abnormal cholesterol or triglyceride levels are most likely associated with dietary factors or hepatic dysfunction. The rabbit has low biliverdin reductase activity^50^ and only 30% of biliverdin is converted to bilirubin. Bilirubin values can be affected by fasting. Atropine-medetomidine-propofol anesthesia raised blood triglycerides levels in dogs as well as increasing total bilirubin in dogs,^49^ but these parameters remained unaffected during the current study. A significant decrease in the value of total bilirubin was seen in group 2. All blood biochemical parameters remained within the physiological limits in rabbits of this study under propofol anesthesia using either IV or IO route. Similar results were reported by Brzeski *et al*. in sheep with IV propofol anesthesia.^36^ As no significant adverse effect was observed with IO injection of propofol on the physiological, hematological and biochemical parameters, the use of IO propofol could be recommended as a valuable and safe method of anesthesia in small animals with limited vascular access. However, caution should be taken on other aspects of IO anesthesia with propofol. For example, the effects of IO propofol on bone marrow are still unknown. In this regard, the clinico-pathologic and pathologic studies are underway (by authors) to determine whether there is any adverse effect.
